# Exceptional Liver Transplant Indications: Unveiling the Uncommon Landscape

**DOI:** 10.3390/diagnostics14020226

**Published:** 2024-01-21

**Authors:** Teodor Cabel, Cristina Madalina Pascu, Catalin Stefan Ghenea, Bogdan Florin Dumbrava, Deniz Gunsahin, Andreea Andrunache, Livia-Marieta Negoita, Afrodita Panaitescu, Ecaterina Mihaela Rinja, Christopher Pavel, Oana-Mihaela Plotogea, Madalina Stan-Ilie, Vasile Sandru, Mariana Mihaila

**Affiliations:** 1 Department of Gastroenterology, Clinical Emergency Hospital of Bucharest, 014461 Bucharest, Romania; cabelteodor@gmail.com (T.C.); denizgunsahin97@gmail.com (D.G.); negoita.livia@yahoo.com (L.-M.N.); ecaterina.rinja@gmail.com (E.M.R.);; 2 Department of Internal Medicine, Fundeni Clinical Institute, 022328 Bucharest, Romaniam.mihaila14@gmail.com (M.M.); 3 Department of Gastroenterology, “Sf. Ioan” Emergency Hospital, 014461 Bucharest, Romania; 4 Department 5, “Carol Davila” University of Medicine and Pharmacy, 050447 Bucharest, Romania

**Keywords:** liver transplantation (LT), end-stage liver disease (ESLD), rare indications (RI), liver disease management (LDM)

## Abstract

Liver transplantation represents the definitive intervention for various etiologies of liver failure and encompasses a spectrum of rare indications crucial to understanding the diverse landscape of end-stage liver disease, with significantly improved survival rates over the past three decades. Apart from commonly encountered liver transplant indications such as decompensated cirrhosis and liver cancer, several rare diseases can lead to transplantation. Recognition of these rare indications is essential, providing a lifeline to individuals facing complex liver disorders where conventional treatments fail. Collaborative efforts among healthcare experts lead not only to timely interventions but also to the continuous refinement of transplant protocols. This continued evolution in transplant medicine promises hope for those facing diverse and rare liver diseases, marking a paradigm shift in the landscape of liver disease management.

## 1. Introduction

Liver transplantation (LT) represents the elective treatment for all etiologies of liver failure [1,2]. The survival rates after LT have improved considerably in the last 30 years, achieving up to 94% one year after transplantation and 61% ten years after [3,4,5,6]. In centers with experience, this surgical procedure is performed routinely with favorable results and low mortality and morbidity rates [7]. Due to effective immunosuppression, improvement of surgical technique, and early diagnosis and management of post-transplant complications, the survival rate has increased significantly [2].

The most common indications for LT are decompensated cirrhosis and liver cancer, followed by acute liver failure. Other indications of LT are represented by metabolic and cholestatic diseases with liver involvement but with lower prevalence [8] (Table 1). 

Besides those common causes of LT, several rare diseases can lead to transplantation [9]. A rare disease is defined as when the incidence is below 1 per 10,000 [10]. Depending on the nature of the disease, rare causes can be neoplastic and non-neoplastic. This analysis focused on the non-neoplastic causes and opportunity of liver transplantation for these infrequent diseases in the usual medical practice and their heartening therapeutic solutions.

## 2. Rare Liver Transplants Indications

### 2.1. Hepatic Hemangiomatosis

Hepatic hemangiomas are among the most prevalent primary liver tumors classified as non-epithelial lesions [11]. Diffuse hepatic hemangiomatosis (DHH) is a rare, most frequently benign condition in which there is widespread substitution of liver tissue by hemangiomatous tumors and usually occurs in newborns [12]. 

The literature review by Shihua He et al. revealed that the most commonly reported symptoms were upper abdominal pain, dyspeptic symptoms, early satiety followed by dyspnea, and systemic symptoms such as high temperatures, excessive night sweats, and fatigue [13,14]. Physical examination findings consist of hepatomegaly in the majority of cases, together with jaundice, peripheral edema, and ascites [15,16,17].

The literature search revealed that DHH has an unclear prognosis due to its low incidence [18].

Surgery is not usually recommended if the margins of the tumor are unclear and there is a risk of bleeding [19]. Four people underwent right/left hepatectomy according to the available studies [17,20,21,22]. During follow-up, only one patient who underwent left hepatectomy was found to have tumor recurrence 6 years later after wedge biopsies [21]. 

Several research studies have described liver transplantation as a viable treatment option for patients with hepatic hemangiomatosis complicated with painful hepatomegaly, liver failure, portal hypertension, or heart failure due to hyperdynamic flow [23,24,25,26,27,28,29]. In a recent systematic literature review by Bhardwaj et al. (September 2022), two patients with DHH underwent living donor transplantation [29] and orthotopic liver transplantation [27], respectively, with good status after a follow-up period of at least 6 months [18].

Trans-arterial embolization, radiofrequency ablation, and radiation have been studied with various rates of effectiveness [28,30,31,32,33]. Studies found that radiotherapy was successful in only one in two patients, with the tumor disappearing three years later [13,34]. Radiotherapy could represent a viable therapy for DHH, but further studies are needed [13]. 

In addition, a therapy with anti-VEGF (anti-vascular endothelial growth factor) has been described, which was successful in liver tumors but had a negative effect on spleen lesions and led to the death of the patient [35].

The most prevalent complication of DHH is liver failure [16,22,36,37]. In reported cases, other complications such as Kasabach–Merritt syndrome, disseminated intravascular coagulation, and cardiac or various organic dysfunctions have also been noted, invariably leading to exitus [13,35].

The literature search showed that radiation could be an alternative treatment, but the last resort is liver transplantation [13].

### 2.2. Amyloidosis

Amyloidosis is a systemic disease that commonly affects a wide variety of organs and can therefore lead to multiple organ failure and ultimately death. Amyloidosis can be classified as primary or secondary due to other diseases such as rheumatoid arthritis or multiple myeloma. Hepatic amyloidosis may be present in a wide variety of patients, but signs and symptoms caused by liver damage may rarely be seen [38]. 

The gastrointestinal tract and the liver are often affected by systemic amyloidosis, besides the most frequent localizations such as heart, kidney, and peripheral nervous system. It is very important to correctly diagnose this systemic disease because it can mimic other GI diseases, thus increasing the challenges for both the patient’s life and the diagnostic process [39]. The clinical and paraclinical manifestations of liver amyloidosis can vary drastically between patients with no symptoms at all or patients with very advanced liver disease. However, severe liver disease manifesting as jaundice and elevated cholestasis are rare, but with a high mortality rate [40]. Usually, the liver is involved in the later stages of amyloidosis only after the involvement of the heart and kidney, and this can represent a sign of poor prognosis for the patient [41].

The main goal of treatment in amyloidosis is to remove or decrease amyloid protein fibrils while maintaining minimal systemic toxicity. Thus, since 1972, various cytoreductive and immunosuppressive drugs have been introduced, as well as drugs and treatment methods used in multiple myeloma. Liver transplantation remains the last resort in the fight against amyloidosis for patients in advanced stages of the disease [42]. A study published in 2009 shows liver response in 69 patients with primary amyloidosis treated with high-dose melphalan chemotherapy and autologous peripheral blood stem cell transplantation. Hepatic response was present in 57% of patients, while hematological response was present in 53% of patients [43].

Hepatic amyloidosis is frequently associated with cardiac amyloidosis and can therefore lead to end stage heart and liver disease [44]. Nardo et al. published a study that included four patients who underwent combined heart and liver transplantation for familial amyloidosis between 1999 and 2003. Therefore, this method, although very difficult to perform, can be a solution for severely affected patients with both cardiac and hepatic amyloidosis [45].

Primary amyloidosis can, in rare cases, present as acute liver failure, as we will further detail through a case from the literature published by M Elnegouly et al. that presents a patient with primary amyloidosis who had been treated with Bortezomib, Dexamethasone, Melphalan, and autologous hematopoietic stem cell transplantation as a bridge to urgent liver transplantation. Eventually, the patient underwent liver transplantation with a very good outcome. Thirty-six months after the orthotopic liver transplantation, the patient is in complete remission [42]. 

The role of liver transplant in amyloidosis is controversial. Some data in the literature suggest that liver transplantation might be useful in patients with dominant hepatic involvement as they are not suitable for chemoimmunotherapy [46]. The first documented liver transplant for transthyretin amyloidosis was performed in 1990 with the purpose of removing the organ that had been producing this abnormal protein. The most favorable outcome from liver transplantation could be found in patients with the Val30Met mutation in the TTR gene [47]. A published study described the experience of liver transplantation in hepatic amyloidosis in a single center. Out of 11 transplanted patients, 6 had improvements in clinical symptoms. The study concluded that liver transplantation is the only therapeutic method for patients in which the liver is the primary site for amyloid protein synthesis [48]. In recent years, the criteria for liver transplantation have become more selective, and the new potential drugs (gene silencers and kinetic stabilizers) have the potential to start a new era in the treatment of transthyretin amyloidosis.

The criteria for liver transplantation have become increasingly stringent in recent years due to advancements in medical therapy such as genetic attenuators and kinetic stabilizers. These advancements have significantly reduced the necessity of liver transplants for amyloidosis. While a new treatment era is emerging to effectively manage this condition, the possibility of transplant remains an alternative option. Future evidence will clarify the benefits for patients and the necessity of liver transplantation in selected cases where medical therapy alone cannot effectively halt the disease progression [49]. 

### 2.3. Sarcoidosis

Sarcoidosis is a multiorgan disease that can therefore affect the hepatic parenchyma. Sarcoidosis is classified as an inflammatory disease. Microscopically, it is characterized by the presence of non-caseating granulomas in the affected organs. The etiology is unknown but a multifactorial etiology is thought to exist, involving genetic, environmental, and immunological causes. Most patients with liver sarcoidosis are asymptomatic. Among patients with systemic sarcoidosis, about 50–70% have specific changes for sarcoidosis in the liver, while only 10–30% of them have altered liver tests [50].

Most frequently, patients present elevated liver cytolysis markers and alkaline phosphatase levels. The diagnosis of sarcoidosis is challenging, with a crucial role attributed to liver biopsy as an indispensable step in confirming and distinguishing this condition [51].

The study by M Sedki et al. analyzed 286 patients with sarcoidosis, of whom only 9.4% also had liver sarcoidosis and only 37% had clinical symptoms. In addition, a quarter of patients with liver sarcoidosis also had cirrhosis at the time of identification of sarcoidosis. The progression of liver sarcoidosis to cirrhosis is highly variable [52].

A study published in 2019 by S Ghoneim et al. included three patients with systemic sarcoidosis and liver sarcoidosis, of whom two patients eventually developed liver cirrhosis. Mortality in sarcoidosis is between 1–5%, not due to liver insufficiency but rather due to cardiac and pulmonary insufficiency and also from the lesions in the central nervous system [53].

Liver transplantation is the last-resort treatment for patients with end-stage liver disease. The most common type of surgery chosen is combined lung and liver transplantation for patients with both pulmonary and liver sarcoidosis [54].

A study published in 2020 by AJ Thuluvath and al. compared patients with sarcoidosis who underwent liver transplantation with patients with primary biliary cirrhosis and with patients with primary sclerosing cholangitis who also underwent liver transplantation between 1985–2016. The patients were compared according to the 5-year survival rate and the graft viability rate. Thus, the study concluded that patients with sarcoidosis undergoing liver transplantation have a lower survival rate, both of themselves and the graft, compared to the other two groups of patients, but approximately 66% of liver-transplanted sarcoidosis patients still had a good survival rate after 5 years of follow-up [55].

Between 1988 and 2004, EJ Lipson and al. assessed 2117 patients who underwent liver transplantation, and hepatic sarcoidosis was an indication in only seven patients (about 0.3%). The survival rate at 1 year for patients and grafts was 100%, and at 5 years, the survival rate was 86%. These survival rates are good and comparable with those of other patients who underwent liver transplantation with other indications. We must keep in mind that end-stage liver disease due to advanced sarcoidosis is a rare indication for liver transplantation when considering all other indications for this specific therapeutic method [56].

The incidence of sarcoidosis tends to be higher in African Americans and white Americans. However, liver failure due to sarcoidosis as an indication for liver transplantation is rare in the United States, accounting for only 0.12% of all liver transplants performed between 1987 and 2007. Although liver failure in sarcoidosis is very rare, the outcome of patients who have undergone liver transplantation is satisfactory. The differences in survival rates between patients with sarcoidosis and other cholestatic diseases (primary biliary cirrhosis and primary sclerosing cholangitis) can be explained by the systemic nature of sarcoidosis and the use of high-dose corticosteroids [57].

### 2.4. Severe Hepatic Trauma

The term hepatic trauma covers a broad spectrum of both blunt and penetrating injuries involving the hepatic parenchyma, blood vessels, and biliary tract, with hepatic trauma accounting for approximately 5% of all trauma [58]. Liver transplantation (LT) completes the spectrum of surgical modalities available in extreme and rare cases of massive lesions, when non-surgical procedures fail to control hemorrhage and complete hepatectomy must be performed [59]. The indications for liver transplantation after liver trauma are limited to uncontrollable bleeding after damage control surgery, extensive liver lacerations that cannot be surgically corrected via hepatectomy, severe lesions of main vascular structures or the main bile duct, and progressive liver failure secondary to trauma with liver necrosis [60].

According to a Brazilian review conducted in 2015 by Ribeiro-Jr et al., of 46 liver transplants in patients with severe liver injury, closed/blunt trauma had a high prevalence with a total of 83%, as well as severe trauma (at least grade IV) with 81%; the main indication in 52% of cases of was acute liver failure, followed by hemorrhage (19%), hepatic necrosis (17%), and biliary fistula (5%) [60]. Another study by Krawczyk et al. in 2016, which included 73 liver transplants after liver trauma, described a 90-day post-transplant mortality rate of 42.5% [61].

The first case of liver transplantation after major liver trauma occurred in 1987, in a patient with complex vascular and biliary injuries after a road accident. In this case, the liver explant was used and the immediate replacement with the donor’s liver was performed in a single step [60]. In 1988, liver transplantation after liver trauma was performed in two stages, hepatectomy followed by termino-lateral porto-cavity and then implantation of the donor’s liver, this technique being used to gain time until a compatible donor is found. Most studies described 36 h of survival in the anhepatic phase, the longest duration being reported at 66 h [62]. In the literature, there is very limited data on LT for liver trauma, which describes a mortality of almost 50% in the first three months post-transplantation [63].

### 2.5. Byler Disease

Progressive familial intrahepatic cholestasis (PFIC) encompasses various types of rare autosomal recessive pediatric liver diseases caused by alterations in genes that impact the transport pathway in hepatocytes. It is characterized by the rapid development of cholestasis accompanied by pruritus and malabsorption, ultimately leading to liver failure [64,65].

PFIC type 1, also known as Byler disease, is caused by defects in the ATP8B1 gene on chromosome 18 (18q21), which produces the FIC1 protein, a “flippase” that protects the structural integrity of the membrane [64,66]. The disease develops in childhood, with cholestasis episodes usually occurring around the third month of life and rapidly progressing to end-stage liver disease, requiring liver transplantation as a last resort [64].

According to the statistical data, PFIC is thought to impact 1 in 50,000–100,000 infants and to warrant 10–15% of pediatric liver transplants [67]. PFIC1 is a relatively rare condition that, along with PFIC2, accounts for two-thirds of all cases of PFIC [68].

In the past, the disorder would typically progress, leading to end-stage liver disease within the first ten years of life. This, along with severe itching and stunted growth, were the primary reasons for undergoing liver transplantation [64,69]. Based on statistical data from the Scientific Registry of Transplant Recipients (SRTR) in the United States, only 101 infants and 24 adults received liver transplants for PFIC1 between 1987 and 2017, accounting for less than 1% of all pediatric liver transplants from 2007 to 2017 [69,70]. Progressive familial intrahepatic cholestasis ranks among the top five prevalent reasons for orthotopic liver transplantation in infants [65].

Due to the systemic nature of PFIC1, liver transplantation does not fully correct the disorder [71]. After transplantation, patients with PFIC1 have reported persistent complications such as refractory diarrhea, pancreatitis, and growth retardation [70,72,73,74]. Additionally, some patients develop progressive fatty liver disease leading to cirrhosis, although the cause of this is still unknown [70,71,72,73,75]. The effectiveness of non-transplant surgical treatments and the risk of graft damage have led to debates regarding the suitability of LT for PFIC [70]. Despite its limitations, liver transplantation remains an effective treatment for children with PFIC1, particularly for those who do not respond to diversion therapy or develop end-stage liver disease with cirrhosis [69,72]. Available data suggests that progressive steatohepatitis is not inevitable and long-term survival can be achieved [69,73]. The rarity of PFIC1 has contributed to a lack of comprehensive long-term outcome data, leading to ongoing controversy regarding the overall benefits of liver transplantation in managing PFIC1 patients [69,74].

### 2.6. Caroli’s Disease

Caroli’s disease (CD) is a rare congenital condition with predominantly multifocal involvement of the biliary tree characterized by segmental dilatation of the medium and large intrahepatic bile ducts [76,77,78]. This corresponds to congenital biliary cysts type V of the Todani classification for cystic biliary disease [79]. Caroli’s disease is characterized by dilatation of the bile ducts without other underlying liver diseases, in contrast to Caroli’s syndrome (CS), which presents intrahepatic bile duct dilations with associated congenital hepatic fibrosis (CHF) and/or polycystic kidney disease (PKD) [80].

Caroli’s disease is rare, with an estimated prevalence of 1 in 1,000,000 individuals [81]. Caroli’s syndrome is more common, affecting 1 in 100,000 people [82]. 

The surgical management remains the only curative treatment [83]. An extremely important aspect of surgical management is determined by the localization of biliary dilations [84]. Depending on this aspect, the involvement can be limited to a single lobe or the entire liver, each of these two forms having a different surgical approach [85].

Liver transplantation is the only curative treatment for Caroli’s disease. LT is indicated in forms where biliary dilatation affects both lobes, causing significant parenchymal damage or recurring episodes of cholangitis [76,86]. In patients with Caroli’s disease with bilobar involvement, liver transplantation is very rare, with the transplant being performed much more frequently in patients with Caroli’s syndrome [87]. 

There are several case series in the literature that describe the experience of different centers that have managed such cases, but no integrative approach has been described up to this point. The reported survival rate is similar to that of those undergoing transplantation for other liver diseases. According to the European Liver Transplant Registry, the survival rate after liver transplantation for Caroli’s disease is 80.9% in a group of 110 patients with Caroli’s disease [87]. 

In a study of 104 patients carried out in the United States of America in which both patients with Caroli’s disease and Caroli’s syndrome were included, the survival rates at 1 year, 3 years, and 5 years, were 86.3%, 78.4%, and 77%, respectively [76]. This study shows a higher survival rate than that reported in Europe, but without separately evaluating the cases of Caroli’s disease from those with Caroli’s syndrome.

Mortality after liver transplantation is not evaluated uniformly in the literature, making it difficult to compare the results. A series of cases from Pittsburgh shows a mortality of 39%, analyzing the situation of 10 transplanted patients, while Millwala, in his study, shows a mortality of 6.3% at 14 days and 8.3% at 30 days after liver transplantation [76,88].

Because liver transplantation is the only curative treatment, with a survival rate similar to that of those who undergo liver transplantation for other diseases, the establishment of a multicentric and multidisciplinary approach to the management of this condition should be considered.

### 2.7. HELLP Syndrome

HELLP syndrome is a severe pregnancy-related condition, occurring in 1 to 6 women per 1000 pregnancies [89]. It is characterized by hemolysis, elevated liver enzymes, and low platelet count, with a high mortality rate of up to 60% [90]. The primary management approach for HELLP syndrome typically involves the prompt delivery of the baby and supportive care for the mother, with a focus on complications such as liver dysfunction [91]. 

Individuals with HELLP syndrome face an increased risk of subcapsular hematoma rupture [89]. While liver transplantation is a crucial intervention for various liver diseases, its specific application for HELLP syndrome is not considered routine.

Within the existing literature, there have been limited instances of liver transplantation for HELLP syndrome, totaling 36 cases [91]. The most common indications for transplantation in these cases include acute liver failure and hemorrhagic shock [92,93,94]. Survival rates vary widely, ranging from 32 days to 12 years, depending on the transplant center [93,95,96,97]. Despite the scarcity of prospective data on post-transplantation evolution and survival, available case series indicate outcomes similar to those observed after transplantation for more common diseases [89]. 

Given the rarity of liver transplantation for HELLP syndrome, there is a pressing need for new studies that offer a more uniform understanding of the progression and survival outcomes associated with this specific condition.

### 2.8. Graft-versus-Host Disease

Graft-versus-host disease (GVHD) is a complication of transplantation with rare incidence. There is an abnormal reaction of the lymphoid tissue of transplanted organ against the receptor tissue [98]. GVHD can appear after any procedure that implies transfers of viable allogeneic lymphocytes [99]. 

Three conditions are involved in the development of GVHD: transfer of immune-competent lymphocytes into a host, the host inability to reject these cells, and an antigenic difference between host and donor tissue [100]. Hepatic GVHD occurs frequently after bone marrow transplant (BMT) and rarely after solid organ transplantation [101]. Incidence of GVHD after liver transplant is estimated at 0.1–2%, with a high mortality rate ranging from 80% to 100%. The most frequent causes of death are infections, gastrointestinal bleeding, bone marrow failure, and multiorgan failure [99]. Because there is currently no widely used clinical or laboratory diagnostic test for GVHD, accurate diagnosis and treatment initiation are frequently delayed [102].

The literature has suggested a number of therapy strategies. According to certain research, immunosuppression can be reduced or even stopped in order to let the recipient’s immune system recover [103]. Other researchers suggest boosting it with corticosteroid medication, thus enhancing basal immunosuppression. However, the death rate is about 75%, and the results are very poor [98]. Unfortunately, GVHD after LT is less responsive to corticosteroids than GVHD after stem cell transplantation (SCT) [99].

Corticosteroids represent the main therapy [102]. The literature discusses instances where corticosteroids were used as a sole form of treatment for patients [99]. Anti-T cell antibodies (Anti-thymocyte globulin, Alefacept) have demonstrated some efficacy in treating corticosteroid-resistant GVHD, but are associated with a higher risk of post-transplant lymphoproliferative illness and viral reactivation [104]. The administration of cytokine inhibitors, most frequently directed against Interleukin 2 (IL2) and tumor necrosis factor α (TNF-α), is the second most popular strategy. Another available treatment choice is Etanercept, a soluble TNF receptor. It is advisable to use fungal prophylaxis alongside this treatment as it often correlates with an increased risk of fungal infections [99,105].

LT is the last treatment option for hepatic GVHD after BMT or non-liver organ transplant [101]. Between 1992 to 2012, the United Network for Organ Sharing (UNOS) reported 112 orthotopic liver transplantations for GVHD with long term survival [106,107]. It is an underutilized modality of treatment and should be considered in steroid refractory hepatic graft-versus-host disease [107]. In these cases, which obtained long-term survival, recurrence of hepatic GHVD has not been reported [101]. The survival rate after LT for hepatic GVHD is inferior to other disease which have indications for LT [106,107].

## 3. Conclusions

Liver transplantation (LT) is the definitive treatment for various liver failures, showing substantial improvements in survival rates over the past three decades. LT is emerging as a routine procedure in experienced centers characterized by low mortality and morbidity rates.

The primary indications for LT include decompensated cirrhosis, liver cancer (especially hepatocellular carcinoma), and acute liver failure, which collectively account for the majority of cases. However, there is a spectrum of rare diseases that lead to the need for transplantation. These rare causes underscore the expanding limits of LT in recent years for uncommon liver diseases. In such exceptional cases, the recommendation for liver transplantation should be brought forth and thoroughly discussed within a multidisciplinary committee. This discussion is crucial not only for pinpointing the most opportune moment for transplantation but also for devising a comprehensive management strategy tailored to the unique circumstances of each individual case.

Liver transplantation is a highly successful intervention for a wide range of liver failures, both common and rare. There is demanding need for the exploration and application of LT in rare non-neoplastic diseases, thus providing hope and viable treatment pathways for patients facing uncommon conditions.

## Figures and Tables

**Table 1:** Rare liver transplant indications.

Disease
Hepatic hemangiomatosis
Amyloidosis
Sarcoidosis
Severe hepatic trauma
Byler Disease
Caroli’s disease
HELLP syndrome
Graft-versus-host disease

## Data Availability

Not applicable.

## References

[1] Galle P.R., Forner A., Llovet J.M., Mazzaferro V., Piscaglia F., Raoul J.-L., Schirmacher P., Vilgrain V. (2018). EASL Clinical Practice Guidelines: Management of hepatocellular carcinoma. J. Hepatol..

[2] Jackson W.E., Malamon J.S., Kaplan B., Saben J.L., Schold J.D., Pomposelli J.J., Pomfret E.A. (2022). Survival Benefit of Living-Donor Liver Transplant. JAMA Surg..

[3] Millson C., Considine A., Cramp M.E., Holt A., Hubscher S., Hutchinson J., Jones K., Leithead J., Masson S., Menon K. (2020). Adult liver transplantation: A UK clinical guideline—Part 1: Pre-operation. Frontline Gastroenterol..

[4] de Ville de Goyet J., Baumann U., Karam V., Adam R., Nadalin S., Heaton N., Reding R., Branchereau S., Mirza D., Klempnauer J.L. (2022). European Liver, Intestine Transplant Association. European Liver Transplant Registry: Donor and transplant surgery aspects of 16,641 liver transplantations in children. Hepatology.

[5] Kwong A.J., Ebel N.H., Kim W.R., Lake J.R., Smith J.M., Schladt D.P., Skeans M.A., Foutz J., Gauntt K., Cafarella M. (2022). OPTN/SRTR 2020 Annual Data Report: Liver. Am. J. Transpl..

[6] Bolarín J.M., Pérez-Cárceles M.D., Hernández Del Rincón J.P., Luna A., Minguela A., Muro M., Legaz I. (2021). Causes of Death and Survival in Alcoholic Cirrhosis Patients Undergoing Liver Transplantation: Influence of the Patient’s Clinical Variables and Transplant Outcome Complications. Diagnostics.

[7] Millson C., Considine A., Cramp M.E., Holt A., Hubscher S., Hutchinson J., Jones K., Leithead J., Masson S., Menon K. (2020). Adult liver transplantation: UK clinical guideline—Part 2: Surgery and post-operation. Frontline Gastroenterol..

[8] Chu K.K., Wong K.H., Chok K.S. (2021). Expanding Indications for Liver Transplant: Tumor and Patient Factors. Gut Liver..

[9] European Association for the Study of the Liver (2016). EASL Clinical Practice Guidelines: Liver transplantation. J. Hepatol..

[10] Finotti M., Auricchio P., Vitale A., Gringeri E., Cillo U. (2021). Liver transplantation for rare liver diseases and rare indications for liver transplant. Transl. Gastroenterol. Hepatol..

[11] Leon M., Chavez L., Surani S. (2020). Hepatic hemangioma: What internists need to know. World J. Gastroenterol..

[12] Batista A., Matos A.P., Neta J.O., Ramalho M. (2014). Diffuse Hepatic Hemangiomatosis in the Adult without Extra-hepatic Involvement: An Extremely Rare Occurrence. J. Clin. Imaging Sci..

[13] He S., Chen W., Yang Y., Tang X., Zhou G., Zhou J., Wu C. (2022). Adult diffuse hepatic hemangiomatosis: A case report and review of the literature. Clin. Res. Hepatol. Gastroenterol..

[14] Rao R., Naidu J., Muhammad Nawawi K.N., Wong Z.Q., Ngiu C.S., Mohammed F., Raja Ali R.A., Yaacob N.Y. (2018). Diffuse hepatic haemangiomatosis: A case report and review of literature. Med. J. Malays..

[15] Bakhshi G.D., Shaikh A., Borisa A.D., Alagappan C., Patnaik A., Subnis B.M. (2008). Diffused liver haemangiomatosis in an adult. Bombay Hosp. J..

[16] Kim J.D., Chang U.I., Yang J.M. (2008). Clinical challenges and images in GI. Diffuse hepatic hemangiomatosis involving the entire liver. Gastroenterology.

[17] Ohkura Y., Hashimoto M., Lee S., Sasaki K., Matsuda M., Watanabe G. (2014). Right hepatectomy for giant cavernous hemangioma with diffuse hemangiomatosis around Glisson’s capsule. World J. Gastroenterol..

[18] Bhardwaj N., Parkhi M., Kumar M., Kaman L., Mitra S. (2022). Adult diffuse hepatic hemangiomatosis. Autops. Case Rep..

[19] Haitjema T., Westermann C.J., Overtoom T.T., Timmer R., Disch F., Mauser H., Lammers J.W. (1996). Hereditary hemorrhagic telangiectasia (Osler-Weber-Rendu disease): New insights in pathogenesis, complications, and treatment. Arch. Intern. Med..

[20] Moon W.S., Yu H.C., Lee J.M., Kang M.J. (2000). Diffuse hepatic hemangiomatosis in an adult. J. Korean Med. Sci..

[21] Lehmann F.S., Beglinger C., Schnabel K., Terracciano L. (1999). Progressive development of diffuse liver hemangiomatosis. J. Hepatol..

[22] Ota T., Kamiyama T., Kato T., Hanamoto T., Hirose K., Otsuka N., Matsuoka S., Taketomi A. (2020). A rare case of cavernous hemangioma accompanied with diffuse hepatic hemangiomatosis. Surg. Case Rep..

[23] Lerut J.P., Weber M., Orlando G., Dutkowski P. (2007). Vascular and rare liver tumors: A good indication for liver transplantation?. J. Hepatol..

[24] Lerut J.P., Orlando G., Adam R., Schiavo M., Klempnauer J., Mirza D., Boleslawski E., Burroughs A., Sellés C.F., Jaeck D. (2007). The place of liver transplantation in the treatment of hepatic epitheloid hemangioendothelioma: Report of the European liver transplant registry. Ann. Surg..

[25] Roque Ramos L., Coelho M.L. (2014). Hepatic haemangiomatosis: Multinodular liver in an asymptomatic elderly man. BMJ Case Rep..

[26] Keegan M.T., Kamath G.S., Vasdev G.M., Findlay J.Y., Gores G.J., Steers J.L., Plevak D.J. (2001). Liver transplantation for massive hepatic haemangiomatosis causing restrictive lung disease. Br. J. Anaesth..

[27] Shankar S., Rammohan A., Kulaseharan V.H., Kanagavelu R., Reddy M.S., Rela M. (2021). Liver Transplantation for Rapidly Progressive Giant Hepatic Hemangioma With Diffuse Hemangiomatosis. Exp. Clin. Transplant..

[28] Hoekstra L.T., Bieze M., Erdogan D., Roelofs J.J., Beuers U.H., van Gulik T.M. (2013). Management of giant liver hemangiomas: An update. Expert Rev. Gastroenterol. Hepatol..

[29] Lee J.H., Yoon C.J., Kim Y.H., Han H.S., Cho J.Y., Kim H., Jang E.S., Kim J.W., Jeong S.H. (2018). Living-donor liver transplantation for giant hepatic hemangioma with diffuse hemangiomatosis in an adult: A case report. Clin. Mol. Hepatol..

[30] Jhaveri K.S., Vlachou P.A., Guindi M., Fischer S., Khalili K., Cleary S.P., Ayyappan A.P. (2011). Association of hepatic hemangiomatosis with giant cavernous hemangioma in the adult population: Prevalence, imaging appearance, and relevance. AJR. Am. J. Roentgenol..

[31] Chalh O., Billah N.M., Nassar I. (2022). Diffuse Hepatic Hemangiomatosis with Giant Cavernous Hemangioma in an Adult. J. Belg. Soc. Radiol..

[32] Malagari K., Alexopoulou E., Dourakis S., Kelekis A., Hatzimichail K., Sissopoulos A., Delis S., Letsou D., Kelekis D. (2007). Transarterial embolization of giant liver hemangiomas associated with Kasabach-Merritt syndrome: A case report. Acta Radiol..

[33] Biswal B.M., Sandhu M., Lal P., Bal C.S. (1995). Role of radiotherapy in cavernous hemangioma liver. Indian J. Gastroenterol. Off. J. Indian Soc. Gastroenterol..

[34] Maeda H., Matsuo T., Nagaishi T., Ikeda T., Tomonaga Y., Mori H. (1981). Diffuse hemangiomatosis, coagulopathy and microangiopathic hemolytic anemia. Acta Pathol. Jpn..

[35] Shimizu Y., Komura T., Seike T., Omura H., Kumai T., Kagaya T., Ohta H., Kawashima A., Harada K., Kaneko S. (2018). A case of an elderly female with diffuse hepatic hemangiomatosis complicated with multiple organic dysfunction and Kasabach-Merritt syndrome. Clin. J. Gastroenterol..

[36] Langner C., Thonhofer R., Hegenbarth K., Trauner M. (2001). Diffuse Hämangiomatose von Leber und Milz beim Erwachsenen [Diffuse hemangiomatosis of the liver and spleen in an adult]. Der. Pathol..

[37] Militz M., Carlotto J.R., Schmitz L.D., Dal Vesco J.A. (2016). Diffuse Hepatic Hemangiomatosis of rapid growth after Bariatric Surgery. Dig. Liver Dis. Off. J. Ital. Soc. Gastroenterol. Ital. Assoc. Study Liver.

[38] Shin Y.M. (2011). Hepatic amyloidosis. Korean J. Hepatol..

[39] Rosenzweig M., Kastritis E. (2020). Liver and Gastrointestinal Involvement: Update. Hematol. Oncol. Clin. North. Am..

[40] Misiakos E.P., Bagias G., Tiniakos D., Roditis K., Zavras N., Papanikolaou I., Tsirigotis P., Liakakos T., Machairas A. (2015). Primary Amyloidosis Manifesting as Cholestatic Jaundice after Laparoscopic Cholecystectomy. Case Rep. Surg..

[41] Lovat L.B., Persey M.R., Madhoo S., Pepys M.B., Hawkins P.N. (1998). The liver in systemic amyloidosis: Insights from 123I serum amyloid P component scintigraphy in 484 patients. Gut.

[42] Elnegouly M., Specht K., Zoller H., Matevossian E., Bassermann F., Umgelter A. (2016). Liver transplantation followed by autologous stem cell transplantation for acute liver failure caused by AL amyloidosis. Case report and review of the literature. Ann. Hepatol..

[43] Girnius S., Seldin D.C., Skinner M., Finn K.T., Quillen K., Doros G., Sanchorawala V. (2009). Hepatic response after high-dose melphalan and stem cell transplantation in patients with AL amyloidosis associated liver disease. Haematologica.

[44] Beal E.W., Mumtaz K., Hayes D., Whitson B.A., Black S.M. (2016). Combined heart-liver transplantation: Indications, outcomes and current experience. Transpl. Rev..

[45] Nardo B., Beltempo P., Bertelli R., Montalti R., Vivarelli M., Cescon M., Grazi G.L., Salvi F., Magelli C., Grigioni F. (2004). Combined heart and liver transplantation in four adults with familial amyloidosis: Experience of a single center. Transplant. Proc..

[46] Theodorakakou F., Fotiou D., Dimopoulos M.A., Kastritis E. (2020). Solid Organ Transplantation in Amyloidosis. Acta Haematol..

[47] Benson M.D. (2013). Liver transplantation and transthyretin amyloidosis. Muscle Nerve..

[48] Singer R., Mehrabi A., Schemmer P., Kashfi A., Hegenbart U., Goldschmidt H., Schönland S., Kristen A., Dengler T., Müller-Schilling M. (2005). Indications for liver transplantation in patients with amyloidosis: A single-center experience with 11 cases. Transplantation.

[49] Ando Y., Adams D., Benson M.D., Berk J.L., Planté-Bordeneuve V., Coelho T., Conceição I., Ericzon B.G., Obici L., Rapezzi C. (2022). Guidelines and new directions in the therapy and monitoring of ATTRv amyloidosis. Amyloid.

[50] Tadros M., Forouhar F., Wu G.Y. (2013). Hepatic Sarcoidosis. J. Clin. Transl. Hepatol..

[51] Kumar M., Herrera J.L. (2019). Sarcoidosis and the Liver. Clin. Liver Dis..

[52] Sedki M., Fonseca N., Santiago P., Diaz L., Garcia-Buitrago M., Mirsaeidi M., Levy C. (2019). Hepatic Sarcoidosis: Natural History and Management Implications. Front. Med..

[53] Ghoneim S., Williams S.D. (2019). Hepatic Sarcoidosis: An Uncommon Cause of Cirrhosis. Cureus.

[54] Bleszynski M.S., Punnen S., Desai S., Hussaini T., Marquez V., Yoshida E.M., Jayakumar S., Chartier-Plante S., Segedi M., Scudamore C.H. (2022). Outcomes of liver transplant recipients with high MELD scores: An experience from a Canadian centre. Can. J. Surg..

[55] Thuluvath A.J., Savva Y., Chen P.H. (2020). Poor Graft and Patient Survival After Liver Transplantation in Sarcoidosis. J. Clin. Gastroenterol..

[56] Lipson E.J., Fiel M.I., Florman S.S., Korenblat K.M. (2005). Patient and graft outcomes following liver transplantation for sarcoidosis. Clin. Transplant..

[57] Vanatta J.M., Modanlou K.A., Dean A.G., Nezakatgoo N., Campos L., Nair S., Eason J.D. (2011). Outcomes of orthotopic liver transplantation for hepatic sarcoidosis: An analysis of the United Network for Organ Sharing/Organ Procurement and Transplantation Network data files for a comparative study with cholestatic liver diseases. Liver Transpl..

[58] Tinkoff G., Esposito T.J., Reed J., Kilgo P., Fildes J., Pasquale M., Meredith J.W. (2008). American Association for the Surgery of Trauma Organ Injury Scale I: Spleen, liver, and kidney, validation based on the National Trauma Data Bank. J. Am. Coll. Surg..

[59] Fong Z.V., Patel M.S., Yeh H., Markmann J.F., Vagefi P.A. (2016). Liver transplantation utilizing a severely fractured graft: Every organ counts. Ann. Hepatol..

[60] Ribeiro M.A., Medrado M.B., Rosa O.M., Silva A.J., Fontana M.P., Cruvinel-Neto J., Fonseca A.Z. (2015). Liver Transplantation after Severe Hepatic Trauma: Current Indications and Results. Arq. Bras. Cir. Dig..

[61] Krawczyk M., Grąt M., Adam R., Polak W.G., Klempnauer J., Pinna A., Di Benedetto F., Filipponi F., Senninger N., Foss A. (2016). Liver Transplantation for Hepatic Trauma: A Study From the European Liver Transplant Registry. Transplantation.

[62] Patrono D., Brunati A., Romagnoli R., Salizzoni M. (2013). Liver transplantation after severe hepatic trauma: A sustainable practice. A single-center experience and review of the literature. Clin. Transplant..

[63] Tucker O.N., Marriott P., Rela M., Heaton N. (2008). Emergency liver transplantation following severe liver trauma. Liver Transpl..

[64] Srivastava A. (2014). Progressive familial intrahepatic cholestasis. J. Clin. Exp. Hepatol..

[65] Gunaydin M., Bozkurter Cil A.T. (2018). Progressive familial intrahepatic cholestasis: Diagnosis, management, and treatment. Hepat. Med..

[66] Vitale G., Gitto S., Vukotic R., Raimondi F., Andreone P. (2019). Familial intrahepatic cholestasis: New and wide perspectives. Dig. Liver Dis..

[67] Jacquemin E. (2012). Progressive familial intrahepatic cholestasis. Clin. Res. Hepatol. Gastroenterol..

[68] Davit-Spraul A., Gonzales E., Baussan C., Jacquemin E. (2009). Progressive familial intrahepatic cholestasis. Orphanet J. Rare Dis..

[69] Henkel S.A.F., Salgado C.M., Reyes-Mugica M., Soltys K.A., Strauss K., Mazariegos G.V., Squires R.H., McKiernan P.J., Zhang X., Squires J.E. (2021). Long-term liver transplant outcomes for progressive familial intrahepatic cholestasis type 1: The Pittsburgh experience. Pediatr. Transplant..

[70] McKiernan P.J., Ganoza A., Squires J.E., Squires R.H., Vockley J., Mazariegos G., Soltys K., Sun Q., Sindhi R. (2019). Evolving trends in liver transplant for metabolic liver disease in the United States. Liver Transpl..

[71] Lykavieris P., van Mil S., Cresteil D., Fabre M., Hadchouel M., Klomp L., Bernard O., Jacquemin E. (2003). Progressive familial intrahepatic cholestasis type 1 and extrahepatic features: No catch-up of stature growth, exacerbation of diarrhea, and appearance of liver steatosis after liver transplantation. J. Hepatol..

[72] Oya Y., Sugawara Y., Honda M., Yoshii D., Isono K., Hayashida S., Yamamoto H., Inomata Y. (2017). Living Donor Liver Transplantation for Progressive Familial Intrahepatic Cholestasis Type 1: Two Reported Cases. Transplant. Proc..

[73] Hori T., Egawa H., Takada Y., Ueda M., Oike F., Ogura Y., Sakamoto S., Kasahara M., Ogawa K., Miyagawa-Hayashino A. (2011). Progressive familial intrahepatic cholestasis: A single-center experience of living-donor liver transplantation during two decades in Japan. Clin. Transplant..

[74] Liu Y., Sun L.Y., Zhu Z.J., Wei L., Qu W., Zeng Z.G. (2018). Liver Transplantation for Progressive Familial Intrahepatic Cholestasis. Ann. Transplant..

[75] Milkiewicz P., Wunsch E., Elias E. (2012). Liver transplantation in chronic cholestatic conditions. Front. Biosci. (Landmark Ed.).

[76] Millwala F., Segev D.L., Thuluvath P.J. (2008). Caroli’s disease and outcomes after liver transplantation. Liver Transpl..

[77] Lasagni A., Cadamuro M., Morana G., Fabris L., Strazzabosco M. (2021). Fibrocystic liver disease: Novel concepts and translational perspectives. Transl. Gastroenterol. Hepatol..

[78] Khan M.Z., Kichloo A., El-Amir Z., Shah Zaib M., Wani F. (2020). Caroli Disease: A Presentation of Acute Pancreatitis and Cholangitis. Cureus.

[79] Tuncyurek O., Lomas D.J. (2016). Should Caroli’s disease be in the Todani classification?. Abdom. Radiol..

[80] Zahmatkeshan M., Bahador A., Geramizade B., Emadmarvasti V., Malekhosseini S.A. (2012). Liver transplantation for caroli disease. Int. J. Organ. Transplant. Med..

[81] Kassahun W.T., Kahn T., Wittekind C., Mössner J., Caca K., Hauss J., Lamesch P. (2005). Caroli’s disease: Liver resection and liver transplantation. Experience in 33 patients. Surgery.

[82] Acevedo E., Laínez S.S., Cáceres Cano P.A., Vivar D. (2020). Caroli’s syndrome: An early presentation. Cures.

[83] Fahrner R., Dennler S.G., Inderbitzin D. (2020). Risk of malignancy in Caroli disease and syndrome: A systematic review. World J. Gastroenterol..

[84] Cerwenka H.J.W. (2013). Bile duct cyst in adults: Interventional treatment, resection, or transplantation?. World J. Gastroenterol..

[85] Ulrich F., Pratschke J., Pascher A., Neumann U.P., Lopez-Hänninen E., Jonas S., Neuhaus P. (2008). Long-term outcome of liver resection and transplantation for Caroli disease and syndrome. Ann. Surg..

[86] Akdur A., Kirnap M., Ayvazoglu Soy E.H., Ozcay F., Moray G., Arslan G., Haberal M. (2017). Unusual Indications for a Liver Transplant: A Single-Center Experience. Exp. Clin. Transplant..

[87] De Kerckhove L., De Meyer M., Verbaandert C., Mourad M., Sokal E., Goffette P., Geubel A., Karam V., Adam R., Lerut J. (2006). The place of liver transplantation in Caroli’s disease and syndrome. Transpl. Int..

[88] Habib S., Shakil O., Couto O.F., Demetris A.J., Fung J.J., Marcos A., Chopra K. (2006). Caroli’s disease and orthotopic liver transplantation. Liver Transplant..

[89] Zarrinpar A., Farmer D.G., Ghobrial R.M., Lipshutz G.S., Gu Y., Hiatt J.R., Busuttil R.W. (2007). Liver transplantation for HELLP syndrome. Am. Surg..

[90] Westbrook R.H., Yeoman A.D., Joshi D., Heaton N.D., Quaglia A., O’Grady J.G., Auzinger G., Bernal W., Heneghan M.A., Wendon J.A. (2010). Outcomes of severe pregnancy-related liver disease: Refining the role of transplantation. Am. J. Transplant..

[91] Mazzola A., Magro B., Perdigao F., Charlotte F., Atif M., Goumard C., Scatton O., Conti F. (2021). Acute liver failure and HELLP syndrome: A clinical case and literature review. Clin. Res. Hepatol. Gastroenterol..

[92] Strate T., Broering D.C., Bloechle C., Henschen S., Pothmann W., Hoffmann S., Izbicki J.R., Rogiers X. (2000). Orthotopic liver transplantation for complicated HELLP syndrome. Case report and review of the literature. Arch. Gynecol. Obstet..

[93] Descheemaeker P.N., Compagnon P., Lavoue V., Seguin P., Lechaux D., Renaud-Giono A., Camus C., Meunier B., Malledant Y. (2009). Liver transplantation for subcapsular haematoma during HELLP Syndrome. Ann. Fr. Anesth. Reanim..

[94] Casey L.C., Fontana R.J., Aday A., Nelson D.B., Rule J.A., Gottfried M., Tran M., Lee W.M. (2020). Acute Liver Failure (ALF) in Pregnancy: How Much Is Pregnancy Related?. Hepatology.

[95] Connor K., Rubin R.A., Shrestha R., Johnson M., Sellers M., Butler B. (2009). ABO incompatible liver transplantation as a bridge to treat HELLP syndrome. Gastroenterol. Res. Pract..

[96] Mikolajczyk A.E., Renz J., Diaz G., Alpert L., Hart J., Te H.S. (2017). Massive Hepatic Infarction Caused by HELLP Syndrome. ACG Case Rep. J..

[97] Varotti G., Andorno E., Valente U. (2010). Liver transplantation for spontaneous hepatic rupture associated with HELLP syndrome. Int. J. Gynaecol. Obstet..

[98] Mora L., Alegre F., Rifon J.J., Marti P., Herrero J. (2018). Treatment of graft-versus-host disease with mesenchymal cells as a complication of a liver transplantation. Rev. Esp. Enferm. Dig..

[99] Rogulj I.M., Deeg J., Lee S.J. (2012). Acute graft versus host disease after orthotopic liver transplantation. J. Hematol. Oncol..

[100] Post G.R., Black J.S., Cortes G.Y., Pollack R.B., Wolff D.J., Lazarchick J. (2011). The utility of fluorescence in situ hybridization (FISH) analysis in diagnosing graft versus host disease following orthotopic liver transplant. Ann. Clin. Lab. Sci..

[101] Barshes N.R., Myers G.D., Lee D., Karpen S.J., Lee T.C., Patel A.J., Finegold M., Goss J.A. (2005). Liver transplantation for severe hepatic graft-versus-host disease: An analysis of aggregate survival data. Liver Transpl..

[102] Chaib E., Silva F.D., Figueira E.R., Lima F.R., Andraus W., D’Albuquerque L.A. (2011). Graft-versus-host disease after liver transplantation. Clinics.

[103] Cutler C.S., Koreth J., Ritz J. (2017). Mechanistic approaches for the prevention and treatment of chronic GVHD. Blood.

[104] Jamil M.O., Mineishi S. (2015). State-of-the-art acute and chronic GVHD treatment. Int. J. Hematol..

[105] Murali A.R., Chandra S., Stewart Z., Blazar B.R., Farooq U., Ince M.N., Dunkelberg J. (2016). Graft Versus Host Disease After Liver Transplantation in Adults: A Case series, Review of Literature, and an Approach to Management. Transplantation.

[106] Moini M., Schilsky M.L., Tichy E.M. (2015). Review on immunosuppression in liver transplantation. World J. Hepatol..

[107] Hashmi S., Taner T., Patnaik M., Leise M., Hathcock M., Kremers W.K., Hogan W., Litzow M.R. (2015). Liver transplantation for hepatic graft-versus-host-disease: A United Network for Organ Sharing (UNOS) Database Study. Biol. Blood Marrow Transplant..

